# Advancing nutritional strategies for brain health: Reconciling epidemiologic findings with clinical applicability

**DOI:** 10.1016/j.tjpad.2025.100466

**Published:** 2026-01-01

**Authors:** Hui Guo, Xiongfei Zhao

**Affiliations:** Xianyang Hospital of Yan’an University, No.38 Wenlin Road, Xianyang City, Shanxi, China

**Keywords:** Anti-inflammatory diet, Alzheimer's disease mortality, Dietary assessment, Cognitive health, Nutritional epidemiology

To the Editor,

We read with great interest the recent article titled “Adherence to an anti-inflammatory diet is associated with lower Alzheimer’s disease mortality: A modifiable risk factor in a national cohort” published in *The Journal of Prevention of Alzheimer’s Disease* [1]. This study stands out for its insightful investigation into the relationship between dietary inflammation and Alzheimer’s disease (AD)-specific mortality, using a nationally representative cohort from NHANES. The authors’ identification of a threshold—≥10 % of total energy intake from anti-inflammatory foods—offers a quantifiable, pragmatic dietary target with potential for wide public health impact. Moreover, the stratified findings in men and non-Hispanic White populations point to nuanced interplays between diet, sex, and race in AD outcomes, opening doors to precision nutritional interventions.

Despite these strengths, several methodological limitations—unaddressed in the paper—warrant further discussion and can help refine future studies in this domain. First, while the authors utilized death certificates via the National Death Index to identify AD-specific mortality, this approach is prone to diagnostic misclassification. AD is often underreported or conflated with other dementia subtypes (e.g., vascular or Lewy body dementia) on death certificates, potentially leading to outcome misclassification and bias in hazard ratio estimates [2]. Future studies should consider broader composite dementia outcomes (ICD-10 F00–F03) and apply sensitivity analyses to ensure the robustness of their findings.

Second, the study did not incorporate baseline cognitive status or incident AD diagnosis data. Cognitive impairment may modify dietary behavior or reflect reverse causality—whereby individuals with early cognitive decline change their food intake. The lack of cognitive function covariates limits causal inference. Future analyses should control for cognitive screening measures where available (e.g., CERAD or DSST in NHANES), or apply causal mediation models to explore whether anti-inflammatory diets delay cognitive decline and thereby reduce AD-related mortality [3].

Third, although the study defines anti-inflammatory foods based on nutrient-rich categories, it does not apply a validated inflammatory dietary index such as the Dietary Inflammatory Index (DII) or empirical dietary inflammatory patterns (EDIP) [4,5]. These indices integrate food components with known inflammatory biomarkers and provide better cross-study comparability. Incorporating such indices—or validating the study’s food-based metric against CRP or IL-6 levels—would enhance the study’s reproducibility and precision.

In conclusion, this study provides compelling evidence supporting the protective role of anti-inflammatory dietary patterns in AD-specific and all-cause mortality. It highlights the potential for simple, accessible nutritional interventions to shape neurodegenerative outcomes at the population level. By addressing the aforementioned limitations, future research can build on this promising foundation and guide targeted preventive strategies against Alzheimer’s disease.

## Data availability

Not applicable.

## Funding

None.

## Declarations of AI use

We have not used any AI at all.

## CRediT authorship contribution statement

**Hui Guo:** Methodology, Writing – original draft, Writing – review & editing. **Xiongfei Zhao:** Supervision, Writing – original draft, Writing – review & editing.

## Declaration of competing interest

The authors declare no conflict of interest.
